# A Rare Case of Coccidioidal Vertebral Osteomyelitis Presenting as Extensive Retroperitoneal and Paraspinal Abscess

**DOI:** 10.51894/001c.166017

**Published:** 2026-07-31

**Authors:** Maria Fatima, Ravjot Bhatia, Mohammed Abdul Hafeez, Venkata Anirudh Chunchu

**Affiliations:** 1 DMC - Sinai Grace

## 123

Coccidioidomycosis is a systemic fungal infection caused by Coccidioides species which are soil-dwelling organisms endemic to the southwestern United States and Central and South America. Vertebral osteomyelitis is severe and even rarer manifestations of rare disseminated infections. We present a rare case of disseminated coccidioidomycosis with extensive retroperitoneal, paraspinal, and vertebral involvement in a young, otherwise healthy individual outside the endemic area.

19-year-old male with PMH of asthma presented with one day of drainage from a left flank abscess preceded by three weeks of back and flank pain. He immigrated from Senegal two years prior and had lived in Arizona for nine months before relocating to New-York-City, where he lived at homeless shelter, before moving to Detroit, Michigan.

On presentation, vital signs were normal with labs revealing a normal WBC count and normal lactic acid. CT imaging revealed large, multiloculated left iliopsoas abscess extending along the thoracic spine, with prevertebral fluid collections and anterior vertebral body erosions, concerning for vertebral osteomyelitis. MRI of the thoracic and lumbar spine showed extensive prevertebral abscess with destruction of the anterior vertebral bodies and preservation of intervertebral disc spaces, without evidence of epidural abscess or spinal cord compression.

Antibiotics were initiated, with drainage of the psoas abscess. Blood cultures and aerobic, anaerobic, fungal, and mycobacterial cultures, TB QuantiFERON testing, were initially negative. Cultures from drainage sites grew rare mold. Spinal bone biopsy confirmed infection with Coccidioides species. Given the patient’s clinical stability and absence of neurologic deficits, long-term antifungal therapy with oral itraconazole was initiated.

Vertebral coccidioidomycosis osteomyelitis is a rare manifestation of disseminated infection and frequently presents with imaging features that mimics tuberculosis. Radiographic similarity, combined with initial negative cultures during early stages of disease state, can delay diagnosis.

This Case highlights the importance of thorough exposure history when evaluating culture-negative vertebral osteomyelitis. In our case, even a remote stay within an endemic area puts our young immunocompetent patient at risk. The early recognition and consideration of Coccidioides with diagnosis through fungal cultures, serology, molecular diagnostics, and tissue biopsy are critical to guide appropriate long-term antifungal therapy and prevent neurologic complications and morbidity.

